# Case Report: Rapid resolution of fever after initiation of third-line rescue treatment with upadacitinib for acute severe ulcerative colitis in two young men

**DOI:** 10.3389/fgstr.2025.1626455

**Published:** 2025-08-19

**Authors:** Dan Pinzaru, Martin Kreysing, Tony Lesmeister, Miriam Schwandner, Patrick Michl, Annika Gauss

**Affiliations:** Department of Gastroenterology and Hepatology, University Hospital Heidelberg, Heidelberg, Germany

**Keywords:** acute severe ulcerative colitis, upadacitinib, fever, rescue treatment, case report

## Abstract

**Introduction:**

Acute severe ulcerative colitis (ASUC) is a life-threatening condition in patients with ulcerative colitis with overwhelming systemic inflammation. In case of steroid-refractory courses, the mainstay of therapy is currently infliximab or a calcineurin inhibitor, weighed against colectomy. Recently, Janus kinase (JAK) inhibitors have been shown to result in rapid and persistent responses even in steroid-refractory patients, so that their position in the therapeutic algorithm of ASUC has to be determined. We present—to our best knowledge, for the first time—two cases in which upadacitinib was administered as a third-line rescue therapy in steroid- and infliximab-refractory patients with persistent fever.

**Case presentations:**

A 33- and a 28-year-old man, both newly diagnosed with ulcerative pancolitis, presented with steroid-refractory courses of ASUC. Both suffered from fever with temperatures of >39°C in spite of empirical antibiotic therapy, and infection was carefully excluded. In both, infliximab at 5 mg/kg body weight failed to resolve the fever, and second salvage therapy with upadacitinib 45 mg led to swift resolution of the fever and to overall clinical response. Both patients were under ongoing upadacitinib treatment, and in outpatient surveillance, one of them in steroid-free clinical remission up to his last follow-up one year post treatment initiation, the other one up to his last follow-up four months post treatment initiation.

**Conclusion:**

Upadacitinib seems to be a valuable option even as a second salvage therapy in ASUC. Randomized controlled trials are warranted. However, it has to be kept in mind that ASUC, especially with septic symptoms such as fever, remains a life-threatening condition in which surgery always has to be evaluated, and that multiple overlapping immunosuppressive therapies may cause severe complications, such as infections.

## Introduction

Ulcerative colitis (UC) is a chronic inflammatory bowel disease (IBD) characterized by inflammation extending from the rectum to the more proximal segments of the colon (1). Clinical manifestations and disease progression differ among individuals, ranging from a predominantly inactive course to chronic, treatment-resistant forms requiring surgery. In some cases, the disease leads to complications such as colorectal cancer and even contributes to mortality (2). Among the clinical phenotypes of UC, acute severe ulcerative colitis (ASUC) is the most critical and potentially life-threatening manifestation. It is characterized by systemic inflammation and bloody diarrhea and may occur in up to 25% of patients during the course of their disease (3, 4). According to ECCO guidelines, ASUC is defined by the modified Truelove and Witts criteria, which include the presence of ≥6 bloody stools per day together with at least one marker of systemic inflammation such as tachycardia (>90 bpm), fever (>37.8°C), anemia (Hb <10.5 g/dL), or elevated inflammatory markers (ESR>30 mm/h or CRP >30 mg/L) (5, 6).

ASUC remains a major therapeutic challenge in clinical gastroenterology. Despite advances in IBD management, ASUC still carries significant morbidity and a high risk of colectomy, particularly in patients refractory to initial therapies (4). First-line treatment consists of intravenous corticosteroids, with infliximab or calcineurin inhibitors (e.g., cyclosporine) as standard rescue options in steroid-refractory cases (3, 4). The risk of colectomy for every episode of ASUC is approximately 13% (7). Janus kinase inhibitors (JAKi), particularly tofacitinib, have recently emerged as promising agents for the treatment of ASUC due to their rapid onset of action, oral administration, short half-life, and lack of immunogenicity (8, 9). In the randomized controlled TACOS trial, tofacitinib at a dose of 10 mg thrice daily was compared to placebo over 7 days, while all patients were continued under intravenous corticosteroids (8). In the recently published TRIUMPH trial, a multicenter open-label study, 24 hospitalized patients with steroid-refractory ASUC—a third of them having previously failed anti-TNFα therapy—were administered tofacitinib 10 mg twice daily, resulting in clinical response after a mean of 2.4 days, and plasma CRP concentrations decreased markedly within the first 2 days after tofacitinib initiation (9). Even though upadacitinib, a JAK1-selective JAKi, has been approved for use in moderate-to-severe UC following anti-TNFα failure, its role in treating ASUC remains to be further investigated. Two recent systematic reviews identified less than 100 cases of ASUC patients treated with upadacitinib, mostly after infliximab failure, suggesting that it may be an effective and safe salvage option in this special setting (10, 11). In particular, Gilmore et al. reported favorable outcomes in a small cohort of infliximab-experienced patients with steroid-refractory ASUC treated with upadacitinib, with five out of six patients avoiding colectomy (12). Remarkably, there are just a few reported cases of patients treated with infliximab followed by upadacitinib during the same hospital admission for ASUC. For instance, Berinstein et al. reported only three such cases, none of whom required colectomy (13). Huynh et al. published a single case of a 69-year-old patient with steroid- and partially infliximab-refractory ASUC who responded rapidly to upadacitinib 45 mg and was in clinical, sonographic, and biochemical remission 12 weeks after treatment initiation (14). Very recently, Etchegaray et al. published a case series of sequential rescue therapy with a JAK inhibitor following unsuccessful treatment with intravenous steroids and dose-intensified infliximab in six adult patients with ASUC. Two among the patients had to undergo colectomy, while four achieved steroid-free clinical remission (15).

In this case report, we present—to our best knowledge, for the first time—two patients with ASUC who were successfully treated with upadacitinib following corticosteroid and infliximab failure and who both suffered from body temperatures of >39°C prior to treatment initiation, further supporting its potential utility in this very critical clinical scenario.

## Case descriptions

We present two cases of patients with steroid- and infliximab-refractory ASUC who presented with body temperatures of >39°C due to colitis.

## Case 1

**Figure 1:**
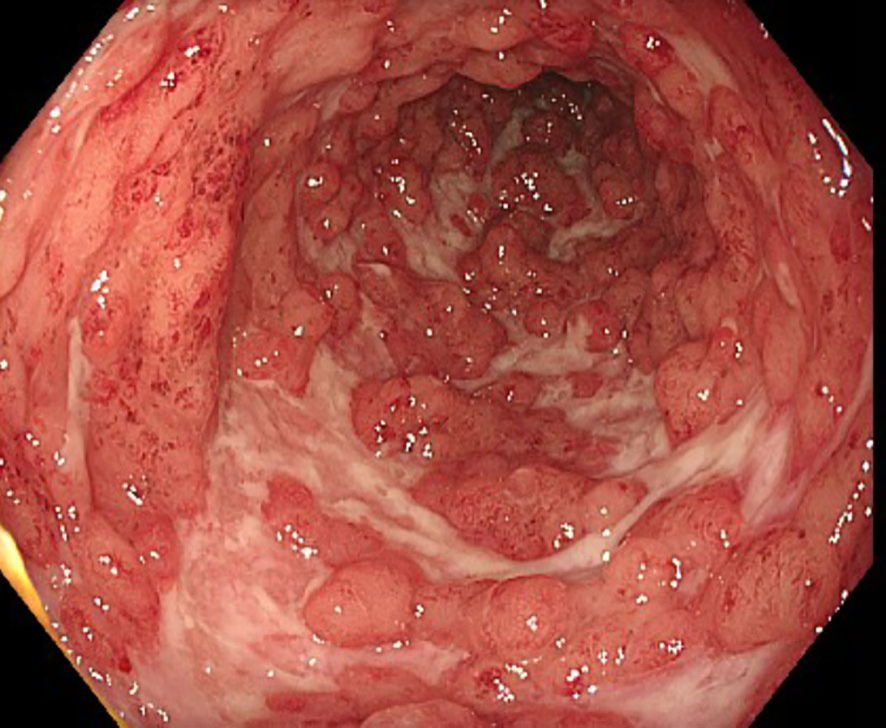
Sigmoidoscopy preceding the initiation of upadacitinib treatment in case 1, showing severe inflammation with deep ulcerations in the sigmoid colon.

**Figure 2:**
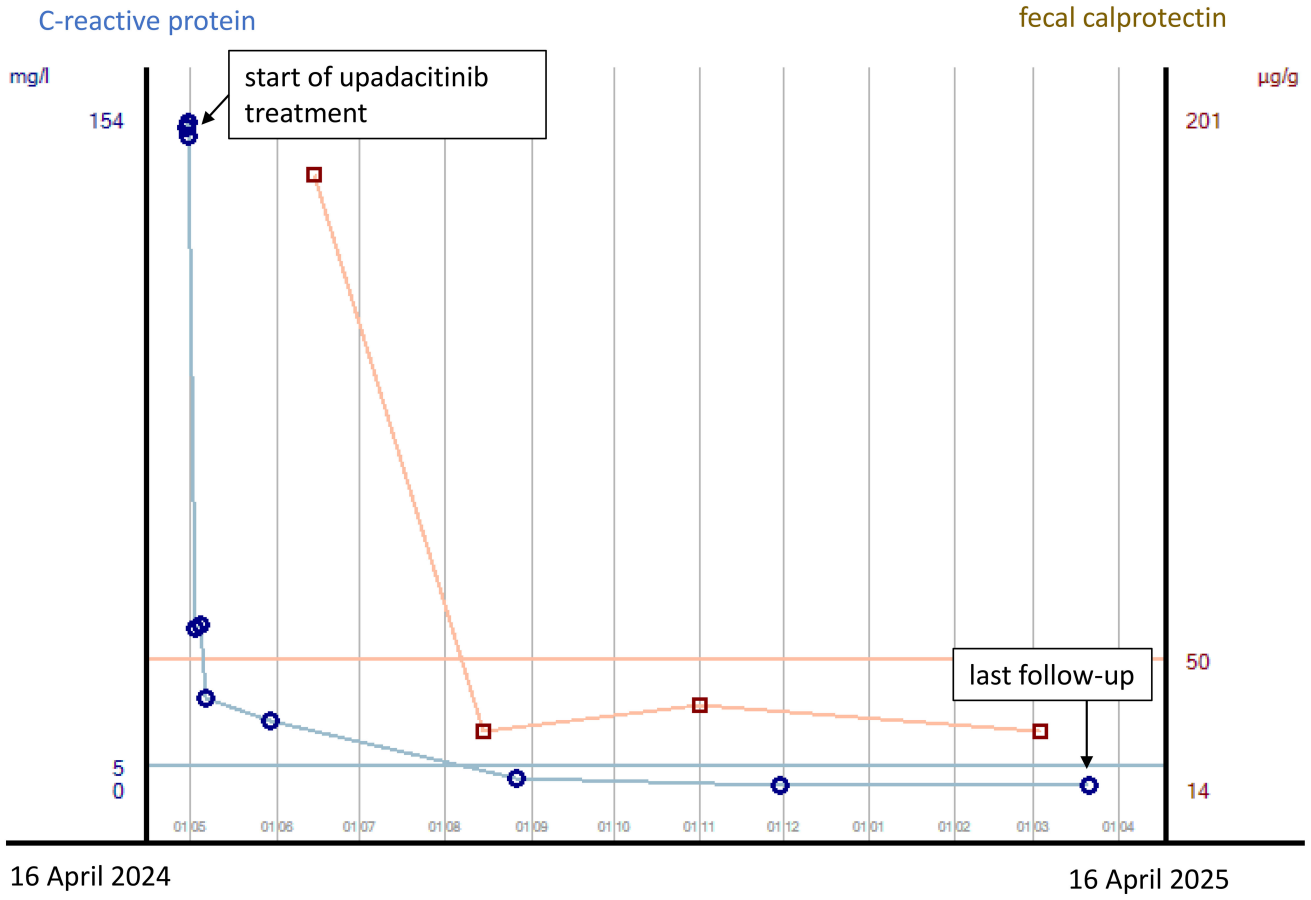
Courses of plasma CRP concentrations and fecal calprotectin concentrations from the start of upadacitinib treatment to last follow-up after 12 months in case 1. CRP concentrations are indicated in mg/L, with concentrations <5 mg/L being normal, and fecal calprotectin concentrations are indicated in µg/g, with concentrations <50 µg/g being normal.

The first patient is a 33-year-old man who was transferred to our tertiary referral center from another clinic in late April 2024 for further inpatient treatment of steroid- and infliximab-refractory ASUC. He reported that the first symptoms had occurred approximately 10 weeks prior, including bloody diarrhea, diffuse abdominal pain, fatigue, and rapid weight loss (15 kg within 3 months). Upon admission to the previous hospital, where he had stayed for 23 days, he had already been suffering from daily body temperature peaks of up to 40°C. He had been diagnosed with ulcerative pancolitis shortly prior to that hospital stay and had been on oral systemic corticosteroids at a dose of 1 mg/kg body weight as well as oral and rectal mesalazine since admission. After an unsuccessful 6-day course of intravenous prednisolone at a dose of 1 mg/kg body weight, the patient had already received one infusion of infliximab at a dose of 5 mg/kg body weight 18 days prior to the transfer to our hospital. This therapy resulted in a slight reduction of stool frequency but no resolution of daily fever. To rule out toxic megacolon and infection as causes of the high fever, the colleagues at the previous hospital had already performed stool, urine, and blood cultures; thoracic X-ray; otorhinolaryngological examination; and echocardiography. A CT scan of the abdomen and thorax showed pancolitis but no signs of toxic megacolon, perforation, or abscedation and no further potential infectious foci in the abdomen and thorax. Cytomegalovirus (CMV) reactivation was excluded by blood and biopsy examinations. Empirical treatment courses with first ampicillin/sulbactam and then piperacillin/tazobactam did not lead to a resolution of the fever. At the patient’s arrival at our center, he reported approximately six bloody bowel movements per day, diffuse abdominal pain, and fever. The patient’s initial body temperature was 39.6°C, his heart rate 110/min, his blood pressure 95/62 mmHg, and his BMI 21.7 kg/m^2^. Physical examination showed slight left-sided abdominal pain without tenderness. Blood laboratory examinations revealed plasma CRP concentration of 153 mg/L (normal: <5 mg/L), hemoglobin concentration of 8.2 g/dL, leukocyte and blood platelet counts of 9.5/nL and 492/nL, respectively, and plasma procalcitonin concentration of 0.26 ng/mL (normal: <0.05 ng/mL). The patient’s medication upon his arrival was oral mesalazine 3 g/day, rectal mesalazine 4 g/day, parenteral nutrition, metamizole 4 g/day, and thromboprophylaxis. An empirical treatment course with piperacillin/tazobactam had been discontinued at the previous hospital, as the fever had persisted under the treatment. Sigmoidoscopy revealed severe inflammation with multiple deep ulcerations (**Figure 1**), and abdominal ultrasound showed pancolitis with bowel wall thickness of up to 5 mm and moderate hyperperfusion. Our first recommendation to the patient was an urgent colectomy. However, the patient denied surgery and wished for a third-line medical salvage therapy. We discussed potential risks thoroughly, especially the risk of severe infections under overlapping and protracted immunosuppressive therapies. We finally decided on upadacitinib 45 mg and started the treatment on the evening of the patient’s transfer. To reduce the risk of complications, we continued thromboprophylaxis with low-molecular-weight heparin and introduced pneumocystis prophylaxis with trimethoprim–sulfamethoxazole. Under the treatment with upadacitinib, the patient’s body temperature decreased to normal by the next day and remained so during the course of treatment. Diarrhea and abdominal pain decreased in parallel. The patient was discharged after 6 days of treatment at our center. Thromboprophylaxis was discontinued. Results of laboratory examinations at discharge were CRP 20.9 mg/L and hemoglobin 10 g/dL. The patient was followed up at our IBD outpatient clinic, where the dose of upadacitinib was reduced to 30 mg/day after 16 weeks of treatment. At his last visit approximately 1 year after upadacitinib treatment initiation, our patient was in steroid-free clinical and biochemical remission with a fecal calprotectin concentration of <30 µg/g. No adverse effects of upadacitinib were reported. Shingles vaccination was recommended, but the costs were not covered by health insurance. Humoral and fecal inflammation parameters in the course of the year are displayed in **Figure 2**.

## Case 2

**Figure 3:**
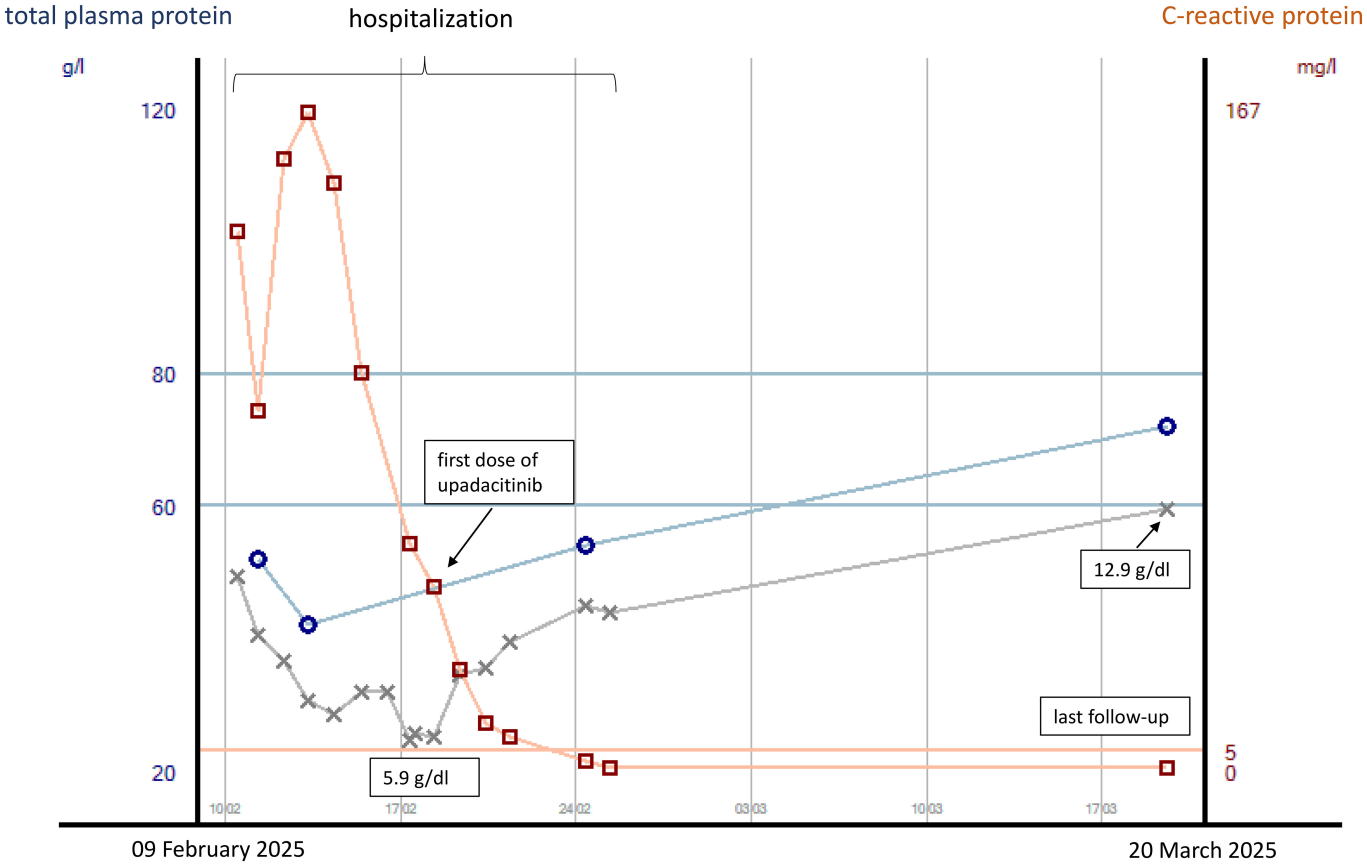
Courses of plasma CRP and total protein concentrations as well as hemoglobin concentrations (gray line) from the start of inpatient treatment to last follow-up in case 2. CRP concentrations are indicated in mg/L, with concentrations <5 mg/L being normal.

Our second patient is a 28-year-old man who presented via the emergency room of our hospital due to profuse watery diarrhea with a stool frequency of approximately 10/day including bloody discharge and clinical signs of exsiccosis. His heart rate was initially 138/min, blood pressure 96/65 mmHg, and body temperature 36.0°C. Blood laboratory results were sodium 125 mmol/L, CRP 136 mg/L, leukocyte count 18.4/nL, hemoglobin concentration 10.9 g/dL, and procalcitonin concentration 0.32 ng/mL. He reported that for 1 month prior to admission, he had been suffering from watery and bloody diarrhea with bowel movement frequencies of approximately 10/day. He had already undergone an outpatient colonoscopy including histological examinations with the new diagnosis of severe ulcerative pancolitis. CMV reactivation and bacterial enteritis had been ruled out. Symptoms had not improved under oral prednisolone at a dose of 1 mg/kg body weight. The patient reported abdominal pain, but the physical examination of the abdomen revealed no tenderness. At his inpatient admission, prednisolone was initially continued intravenously at a daily dose of 1 mg/kg body weight, and the patient received IV fluids and parenteral nutrition, as well as thromboprophylaxis with low-molecular-weight heparin. On day 2 of admission, the patient developed fever with temperatures of up to 39.9°C. Procalcitonin concentration was elevated at 0.58 ng/mL. Empirical antibiotic therapy with ceftriaxone and metronidazole was started on day 3 of admission for fear of bacterial translocation from the bowel. Blood, urine, and stool cultures taken prior to the initiation of antibiotic treatment were all negative. Due to non-response to IV steroids, infliximab was started at a dose of 5 mg/kg body weight on day 5 of admission. Under this treatment, the patient reported slightly less bloody stools with persistently high bowel movement frequencies. Body temperatures decreased first, only to increase again to >39°C 3 days after infliximab infusion. Anemia increased with a minimum hemoglobin concentration of 5.9 g/dL, necessitating blood transfusion 5 days after infliximab infusion. CRP concentrations had started to decrease 1 day after the initiation of antibiotic therapy. Due to the life-threatening condition and inadequate response to infliximab on day 5 (i.e., on day 10 of admission), we discussed colectomy with the patient, also weighing it against the slight chance of success for a second medical salvage therapy with associated risks of potential side effects. The patient wished to try another medication under strict surveillance and standby colectomy. After thorough discussion, especially taking into account the risk of severe infection under extensive immunosuppressive therapy, treatment with upadacitinib was started at a dose of 45 mg/day. Prednisolone had already been switched to hydrocortisone at a dose of 30 mg per day at the first dose of infliximab to reduce the risk of infection. For further risk reduction, the patient received pneumocystis prophylaxis. CRP concentrations decreased to normal by day 7 of upadacitinib initiation. By day 2 of upadacitinib treatment, the bowel movement frequency decreased from 10 to 6 per day, and bloody discharge decreased. The patient was released to outpatient care on day 6 after upadacitinib treatment initiation with three bowel movements per day without bloody discharge. Thromboprophylaxis was discontinued. He presented at our IBD outpatient clinic 4 weeks following upadacitinib treatment initiation with no abdominal pain, no bowel urgency, three bowel movements per day of solid stool consistency without bloody discharge and without urgency, and a body weight gain from 56 to 62.6 kg since his discharge from inpatient treatment. Hemoglobin concentration was 12.9 g/dL, CRP concentration <0.5 mg/L, and total protein concentration 72.0 g/L. Due to the excellent treatment response, the daily dose of upadacitinib was reduced to 30 mg 8 weeks after treatment initiation. At his last visit to our outpatient clinic, 18 weeks after treatment initiation, the patient went on to be in steroid-free clinical remission and had reached his normal body weight of 67 kg. Laboratory parameters were as follows: hemoglobin concentration 15.5 g/dL, CRP concentration <0.5 mg/L, and fecal calprotectin concentration 35 µg/g. So far, the patient has not experienced any side effects of upadacitinib. The courses of plasma CRP and plasma protein concentrations and hemoglobin concentrations during the hospital stay and after discharge are displayed in **Figure 3**.

## Discussion

We report two cases of young male patients with steroid-refractory ASUC in recently diagnosed UC who presented with septic symptoms with high body temperatures of over 39°C and who responded very rapidly to upadacitinib third-line rescue therapy.

Both patients displayed severe disease activity in spite of IV prednisolone treatment at doses of 1 mg/kg body weight. International guidelines recommend either infliximab or cyclosporine in adult patients with steroid-refractory ASUC (6). The decision on the choice of medication should be made according to the center’s experience and the plan for further therapy. Both of our patients received infliximab. However, they showed inadequate response to infliximab therapy, with persistent fever and bloody diarrhea. In our two patients, infliximab was administered at the standard dose of 5 mg/kg body weight, rounded up to 100 mg. It is debatable whether an intensified dose of 10 mg/kg body weight may have resulted in an adequate therapeutic response. However, the recently published open-label randomized controlled PREDICT-UC trial did not reveal superiority of a 10-mg/kg infliximab regimen to 5 mg/kg infliximab in various endpoints including short-term clinical response and colectomy rates (16). Current guidelines recommend that third-line medical treatments in ASUC should be considered very carefully and only be performed in specialized centers, as it has been revealed that they are associated with a high risk of complications as serious infections and may delay surgery to a point where surgery-associated complications increase (6, 16, 17).

Corresponding data have been published mainly for infliximab, cyclosporine, and tacrolimus, while large data sets on JAKi as third-line rescue therapies are not yet available. The reason for which we decided on third-line medical therapies in our patients instead of surgery was mainly based on their strong wish not to undergo surgery and to the fact that upadacitinib has a short half-life and could have been discontinued quickly in case of surgery. The decision against using a calcineurin inhibitor and for a JAKi was made based on published case series, extensive experience with calcineurin inhibitors, and their disadvantages including a narrow therapeutic index, high risk of side effects, and the necessity to switch to a different maintenance therapy in case of success with again uncertainty of therapeutic effects. Recently, Gisbert and Chaparro have published a review on the use of JAKi in the management of ASUC (11). So far, most data on the use of JAKi in ASUC exist on the unselective JAKi tofacitinib. As of yet, 30 studies (including 373 patients) have analyzed the efficacy of tofacitinib in ASUC, resulting in an avoidance of colectomy in 82% (weighted mean) (11). Data on upadacitinib in this treatment situation are more limited but also reveal high mean colectomy-free rates ranging between 67% and 100% (11). No data are available on the use of filgotinib, another JAK1-selective JAKi, in ASUC. Tofacitinib, filgotinib, and upadacitinib have all been approved for the treatment of moderate-to-severe UC (18–20). So far, these medications have not been compared to one another in head-to-head clinical trials. However, there are hints that upadacitinib might be the most efficacious candidate among them (21). Gilmore et al. published six cases of patients who were treated with upadacitinib for steroid-refractory ASUC (12). However, none of them had received infliximab within 8 weeks before the initiation of upadacitinib treatment. One case that is similar to our case was published by Huynh et al. (14). In that case, as in ours, the patient responded to upadacitinib treatment in ASUC after failure of IV steroid and infliximab treatment.

The two patients presented in this case report both suffered from high fevers. Fevers are seen in 40% of IBD patients at the time of presentation. They may occasionally be high but are usually low grade and may stay unrecognized (22). The febrile response is thought to be mediated by endogenous mediators. Among these are pyrogenic cytokines such as tumor necrosis factor (TNF), interleukin (IL)-1, IL-6, and interferons (23). These pyrogenic cytokines belong to the cytokines that play a key role in the pathogenesis of IBD (24). Elevated body temperatures are included as one criterion in the Truelove and Witts’ definition of ASUC (5). There are no structured recommendations on this special situation in patients with ASUC. From a practical, clinical point of view, reluctance to use (combined) immunosuppressive therapies rises with increasing body temperatures, especially if they exceed 39°C. It is of utmost importance to exclude infection as the cause of fever prior to the intensification of immunosuppressive treatment, and toxic megacolon and microperforation must always be considered. Decisions have to be made quickly in ASUC due to disease dynamics. At our center, empiric antibiotic therapy is frequently initiated in parallel to immunosuppressive therapy for ASUC, as bacterial translocation from the bowel is suspected. However, our clinical experience shows that the administration of systemic antibiotics in this situation results in a decrease of plasma CRP concentrations, but not in the improvement of ASUC symptoms such as bloody diarrhea, anemia, fever, and malnutrition. This is how we explain the decrease of CRP levels in case 2 even prior to treatment initiation with upadacitinib, but also steroids and infliximab. The fact that upadacitinib—in contrast to steroids and infliximab—resulted in a rapid resolution of fever in the two reported cases may be explained by the fact that upadacitinib primarily inhibits the JAK–STAT pathway, which is crucial for cytokine signaling (25). Even though serum cytokine levels were not determined in our patients, it may be hypothesized that they had high blood concentrations of pro-inflammatory—and especially pyrogenic cytokines—which were efficiently and quickly inhibited by the action of the JAK inhibitor. It might even be investigated in future studies whether ASUC patients with high fever show better response to upadacitinib therapy than those without.

Many patients who did not have to undergo colectomy in their first hospital stay still have to receive surgery within the year after hospitalization for ASUC. Our case report adds to the body of knowledge on ASUC treatment not only as it describes two patients with fever prior to third-line rescue therapy with upadacitinib but also as our first patient has had a post-hospital stay follow-up of 1 year of clinical remission and biochemical remission with fecal calprotectin concentrations of <30 µg/g so far. This shows that upadacitinib may not only be a short-term rescue treatment but also a long-term solution for some patients. Biomarkers to identify which patients benefit from upadacitinib treatment in ASUC have still to be identified and would be very helpful in this life-threatening situation.

In general, it is important to note that ASUC remains a severe and life-threatening disease. Risk management strategies have to be established, such as standard operating procedures for pneumocystis prophylaxis, thromboprophylaxis, enteral and parenteral nutrition, and the immediate availability of an experienced surgeon.

## Patient perspective

Both patients have reported ongoing clinical remission of their UC. Neither patient regrets his decision to forgo surgery, and they are happy with their treatment. Neither patient has experienced side effects of upadacitinib or suffered from serious infections as of their last follow-up in April (case 1) and June (case 2) 2025.

## Conclusion

In all, we think that it is important to solidify the role of JAK inhibitors, especially of upadacitinib, as second- or third-line treatment in ASUC. Even first-line treatment strategies have to be considered. However, randomized controlled trials are warranted to incorporate these medications into therapy guidelines.

## Data Availability

The data analyzed in this study are subject to the following licenses/restrictions: The datasets are clinical source data acquired in clinical routine and saved in the clinical information system. Insight in the clinical information system by third parties is not allowed for reasons of data protection. Requests to access these datasets should be directed to the corresponding author.
